# Supplementary material for: Pasteurized Milk Serves as a Passive Surveillance Tool for Highly Pathogenic Avian Influenza Virus in Dairy Cattle
Source: Viruses. 2025 Sep 28;17(10):1318. doi: 10.3390/v17101318 (PMC12568113; doi:10.3390/v17101318)
Supplement: Supplementary file 1 [file viruses-17-01318-s001.zip › SupplementaryFigures_revised.pdf]

Supplementary Figure S1. Whole-genome sequencing depth and coverage. Read depth from whole-genome sequencing at each base is plotted for samples S154 (A), S155 (B), and S156 (C). Coverage plots are shown separately for each segment, indicated by the major gene encoded in each segment: nonstructural protein (NS1), matrix protein (MP), neuraminidase (NA), nucleoprotein (NP), hemagglutinin (HA)**,** polymerase acidic protein (PA), polymerase basic 1 protein (PB1), and polymerase basic 2 protein (PB2). Inset: Median read length (MRL) of untrimmed reads mapping to each segment.

A

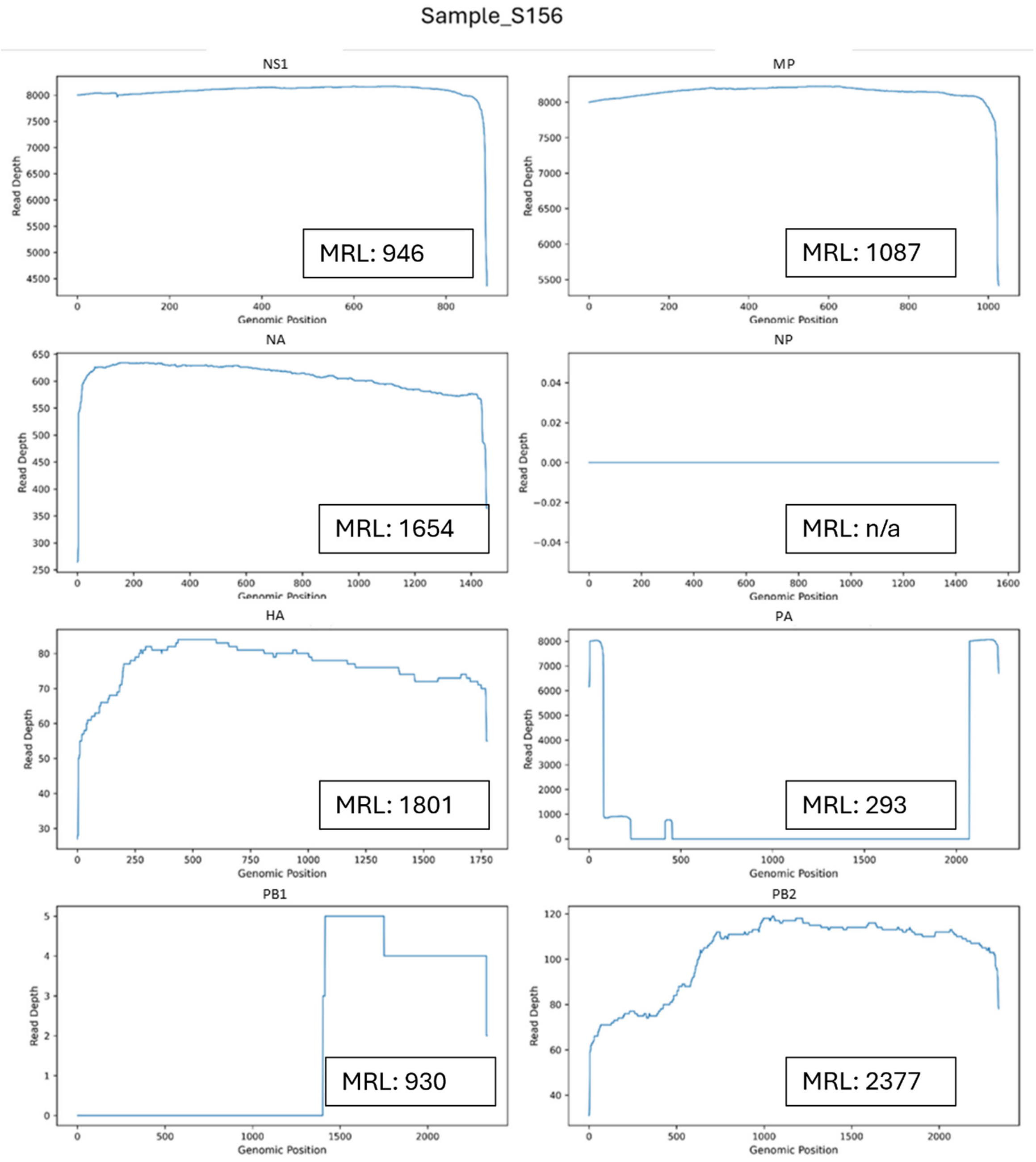

B

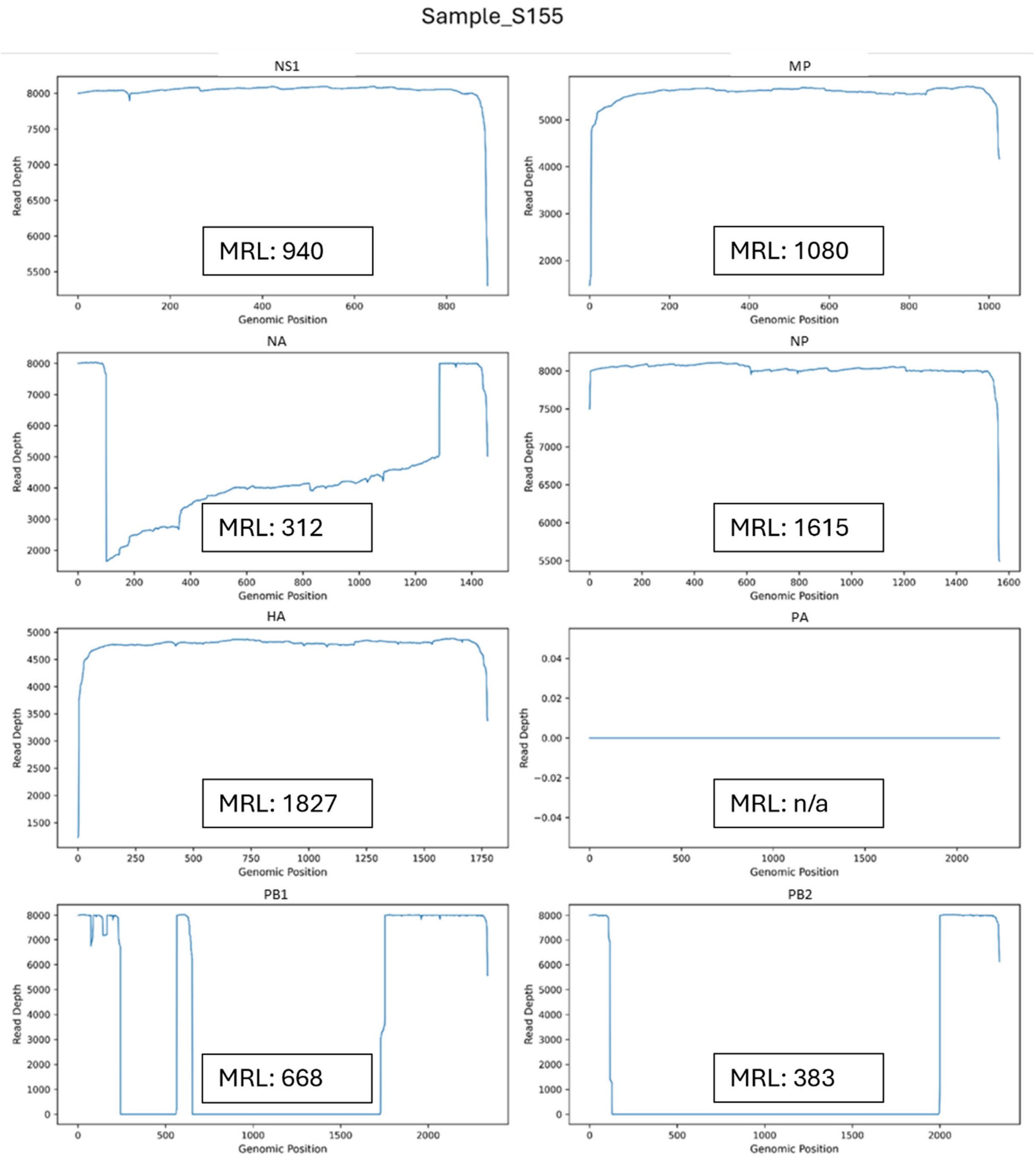
Sample_S155
NS1
MRL: 940
MP
MRL: 1080
NA
MRL: 312
NP
MRL: 1615
HA
MRL: 1827
PA
MRL: n/a
PB1
MRL: 668
PB2
MRL: 383
Read Depth
Genomic Position

C

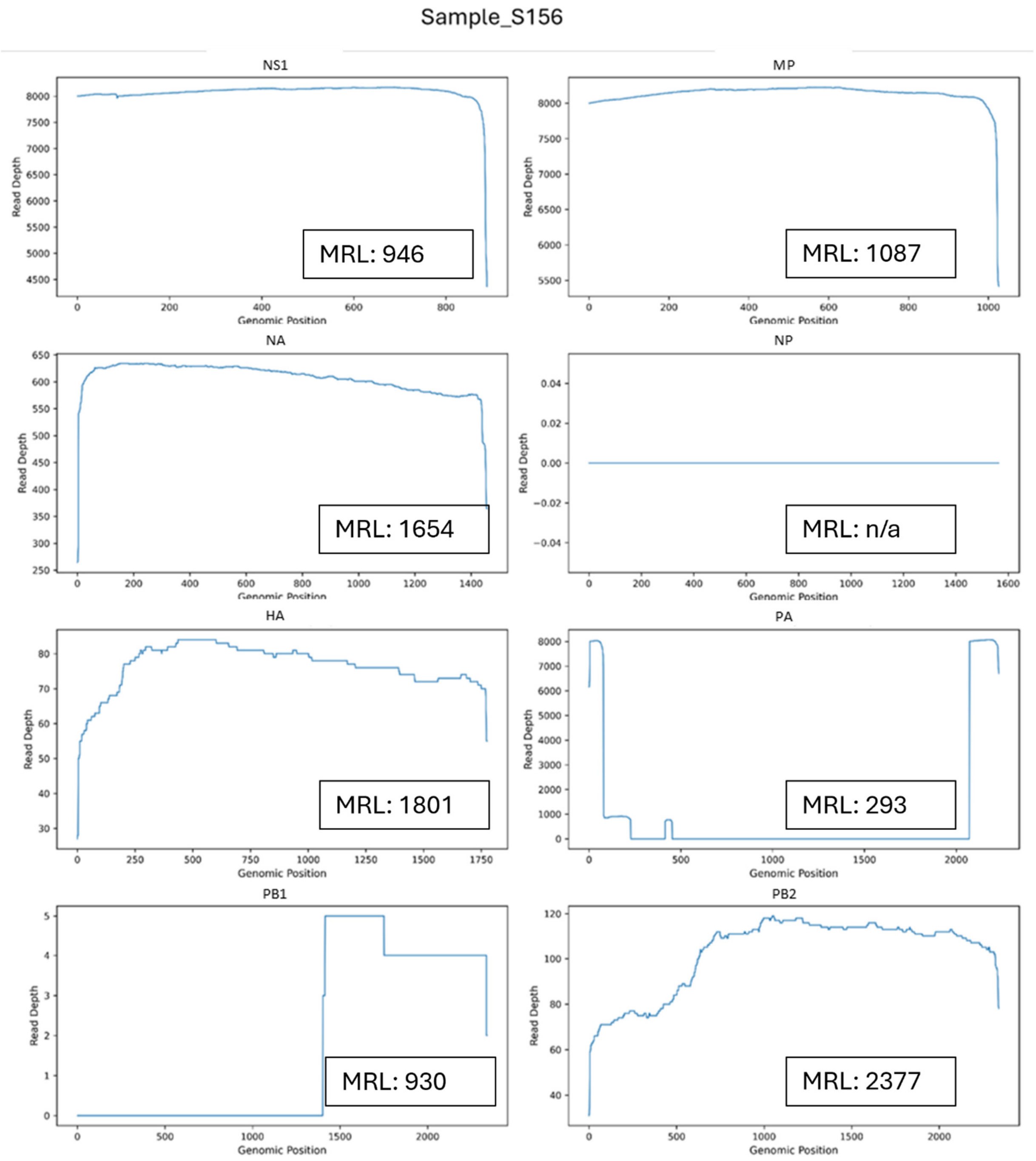
Sample_S156
NS1
MRL: 946
MP
MRL: 1087
NA
MRL: 1654
NP
MRL: n/a
HA
MRL: 1801
PA
MRL: 293
PB1
MRL: 930
PB2
MRL: 2377
Read Depth
Genomic Position

Supplementary Figure S2. Phylogenetic relationship of H5. The H5 gene was amplified as previously described by Suarez *et al* [1]. Amplification products underwent Sanger sequencing at the Penn State Genomics facility. Hemagglutinin (HA) gene sequences of H5 subtype avian influenza viruses were retrieved from the GISAID EpiFlu database for the period from March 1, 2024, to May 31, 2025. Identical sequences were removed using a custom Python script, resulting in a non-redundant dataset of 165 HA nucleotide sequences. Multiple sequence alignment (MSA) was performed using the MAFFT v7.52 [2]. The aligned sequences were trimmed to retain region positions 231 to 731, corresponding to a conserved region of the HA gene. A maximum-likelihood phylogenetic tree was refined using the Nextstrain Augur toolkit (v13.0.0) (https://docs.nextstrain.org/projects/augur/en/7.0.2/). The final tree was exported in JSON format and visualized using Auspice. Tip colors correspond to the geographic origin of sequences.

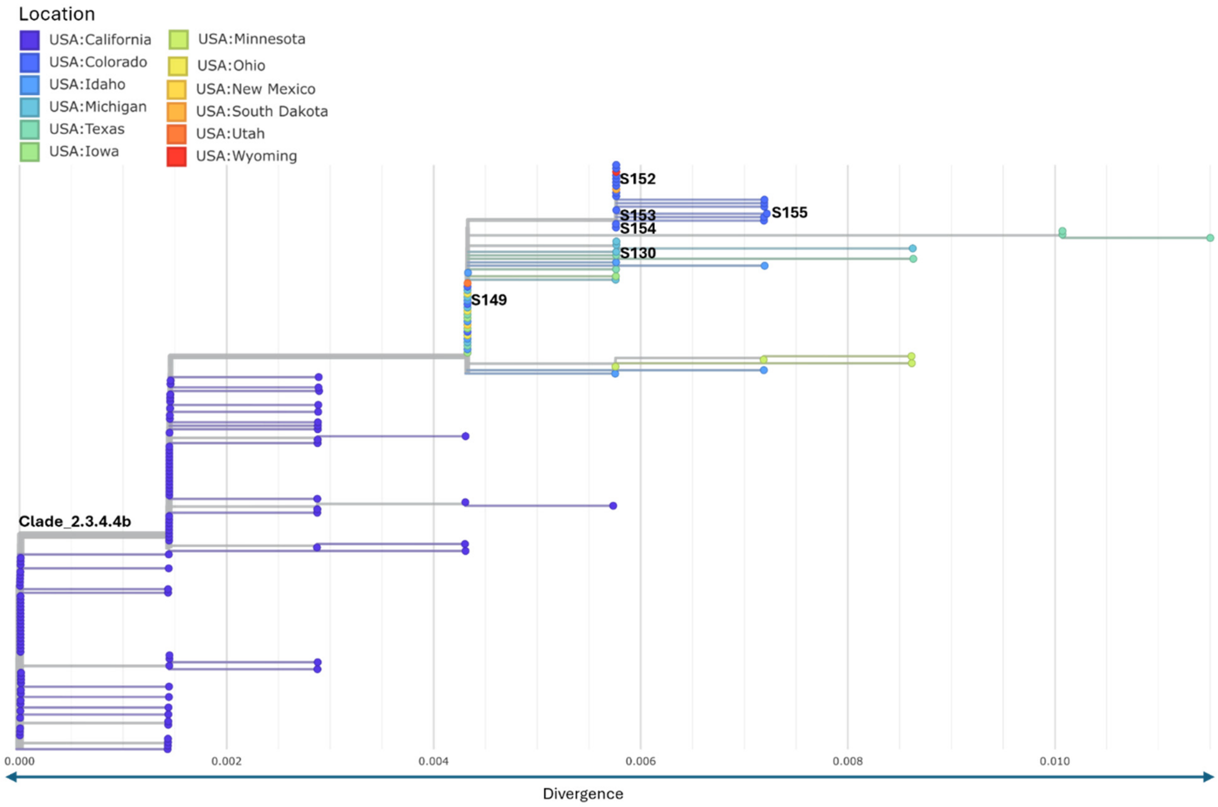

1. Suarez, D.L.; Goraichuk, I. V.; Killmaster, L.; Spackman, E.; Clausen, N.J.; Colonius, T.J.; Leonard, C.L.; Metz, M.L. Testing of Retail Cheese, Butter, Ice Cream, and Other Dairy Products for Highly Pathogenic Avian Influenza in the US. *J Food Prot* **2025**, *88*, 100431, doi:10.1016/j.jfp.2024.100431.

2. Katoh, K.; Standley, D.M. MAFFT Multiple Sequence Alignment Software Version 7: Improvements in Performance and Usability. *Mol Biol Evol* **2013**, *30*, 772–780, doi:10.1093/molbev/mst010.
